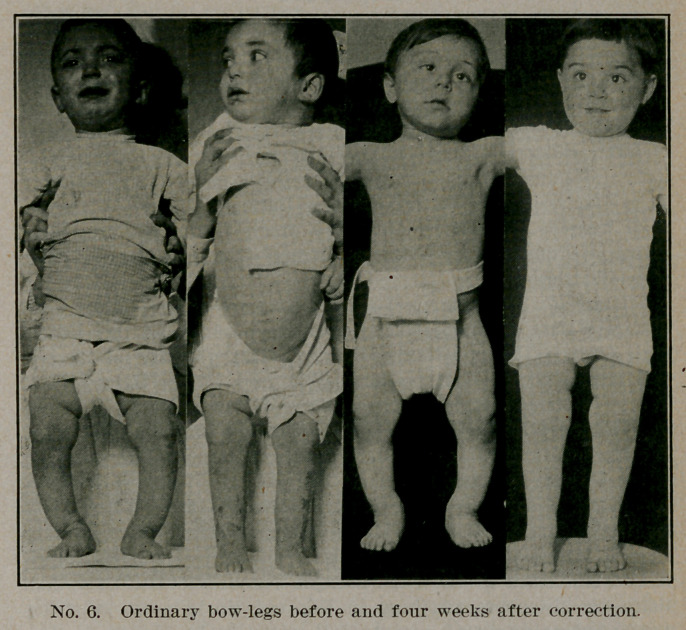# Some Practical Points Concerning the Operative Treatment of Bow-Leg and Knock-Knee

**Published:** 1913-04

**Authors:** Prescott Le Breton

**Affiliations:** Buffalo; Orthopadist to the Erie County and Columbus Hospitals Assistant Orthopadist to the Children’s Hospital Member of the American Orthopadic Association


### Some Practical Points Concerning the Operative Treatment of Bow-leg and Knock-knee.

By PRESCOTT LE BRETON, M.D.
Buffalo
Orthopadist to the Erie County and Columbus Hospitals
Assistant Orthopadist to the Children's Hospital
Member of the American Orthopadic Association

THE common deformities of bow-leg and knock-knee have been well understood for a long time and the treatment,, both mechanical and operative, fairly settled. They form a very satisfactory class, because the results are good and the cases easy to handle. Parents can see for themselves the marked changes in contour, especially when they are presented with photographs of the case before and after. Although the great majority of these cases are due to rickets, there is a goodly proportion where the deformities are due to other conditions, e.g., infantile paralysis, tuberculosis of bone, injuries, congenital deformities, etc.

PLATE NO. 1.

Rickets: Psuedo-paralysis.

In the treatment of these cases by osteoclasis and osteotomy the following facts have impressed the writer in his personal experience. In the first place the wonderful change which these

No. 2. X-ray and standing posture four weeks after correction.

children show when the acute stage of rickets is treated by rest in bed, diet, fresh air and tonics, such as thryoid extract. The transformation in their mental and physical being is often extraordinary. Plate one gives the X-ray of a case of pseudo-paraly-

No. 3. Standing posture one month, and X-ray one year after correction.

sis in which the bone changes were so marked that at first there was some doubt as to whether the lesion was rickets or some more serious disease of the skeleton. Yet this child in the hospital ward improved most rapidly and complete cure resulted from general treatment. Operations are regularly de-

No. 4. X-ray shows site of osteotomy. Note change in nutrition.

ferred until it is evident that nutrition has improved and the bones hardened to a certain extent. If this is not done it is possible for the case to develop, after the correction of one deformity, another deformity, due to weightbearing while the bones are soft.

The best way to get the consent of the parents, who are usually ignorant, is to say that the bones are to be bent straight under a little chloroform, because if an explanation is made in detail as to methods they become frightened. Even trained nurses will show signs of mental disturbance when bones are broken, and yet if the anaesthetist watches while the fracture is effected, he will note, as a rule, no changes in pulse, respiration or pupil.

No. 5. Double osteotomy. Note change in nutrition.

The X-ray is very useful; before operation it shows the lesions and the site of the apex of the curve; afterwards it shows the fracture and illustrates the fact that slight overcorrection at one point will bring the leg into line and result in practical cure.

The ideal at the time of operation is a partial break with bending—the continuity of bone and periosteum being preserved. The manipulation of osteoclast and chisel is a matter learned by practical experience; speed in the manipulation of the oseoclast is important both for pressure and release; proper padding and avoidance of pressure on the epiphyses are essential; the osteotome or chisel can be guided by the sensation of the hand so as to avoid damage to soft tissues.

The method of application of the plaster is important. For bow-legs corrected below the knee the pelvis need not be included, but for any operation above the knee a spica should be used to hold the position. The protective should be snugly applied in just sufficient quantity to allow for a little swelling. If it is applied loosely or too thickly part of the correction may be lost. As the plaster sets the foot should be held at right angles and the rotation altered so as to bring the knee cap in line with the forefoot. After several hours have elapsed the case should be seen again to insure the fact that there is no marked pain

No. 6. Ordinary bow-legs before and four weeks after correction.

or signs of constriction. The only case of pressure paralysis the writer has seen occurred from neglect of this rule. The nurses in this particular case gave paregoric for pain and did not report the condition. The next day the toes were swollen and bluish and it was found that some swelling at the site of the osteotomy had caused constriction. A temporary weakness of the calf muscles followed.

All operators admit that sometimes on removal of the plaster, a deformity, not fully appreciated before, becomes much more evident. For example, after the correction of a knock-knee, some bow-leg deformity appears or vice versa. This may call for a second operation.

The after treatment is of importance. The ordinary rule is for the plaster to be removed in three to four weeks, and the patient to remain in bed another week for massage and light movements. The bow-leg cases especially need changes in the shoes. Stiffened counters, Boston heels, and insoles are indicated to keep the weight-bearing line straight and to prevent intoeing.

---

Eighty-fourth Convention of German Natural Scientists and Physicians.—V. Czerny, Heidelberg. Reported complete in *Zent. fur Chir.*, December 14, 1912. The non-operative treatment of cancer. Czerny delivers a masterful oration on this subject and stimulates the interest in the treatment of non-operable cancers. He gives a summary of the most recent palliative measures which here and there have resulted so favorably as to give hope for the discovery, in the near future, of a sovereign remedy. He speculates very much to the point on how many institutes for cancer research might be built for the price of one battleship.

Should any physician feel at loss for further measures of relief in a case of inoperable cancer, he might read this article with profit. The more expensive measures, that is, the electric and radium treatments, it remains for the hospitals to carry out. We have about one death from cancer for every two physicians in Buffalo each year, to say nothing of the active living cases. We have the right to expect help from the new hospital on High street (which is more than half completed), if not in the discovery of a cure, at least in the dissemination of suggestions leading to greater activity in treatment of inoperable cases, so that no Aesculapian need shrug his shoulders over a case until it ends in death or the ever possible recovery. The only man that ever discovers anything in this old world is he who has found the fountain of perpetual youth, namely, chronic enthusiasm.

---

Use of Caecum to Replace the Extirpated Bladder.—P. Lengemann, Bremen (*Zentralblatt für Chirurgie,* December 14, 1912). Lengemann describes an improvement on the technic of Makkas. He uses the ascending colon as well as the caccum for greater capacity and attaches the ureters to a portion of the ileum, thus utilizing the ileocaecal value to prevent back flow from the artificial bladder. The appendix is brought through the abdominal wall just as in Makkas's procedure.

## Figures and Tables

**Plate No. 1. f1:**
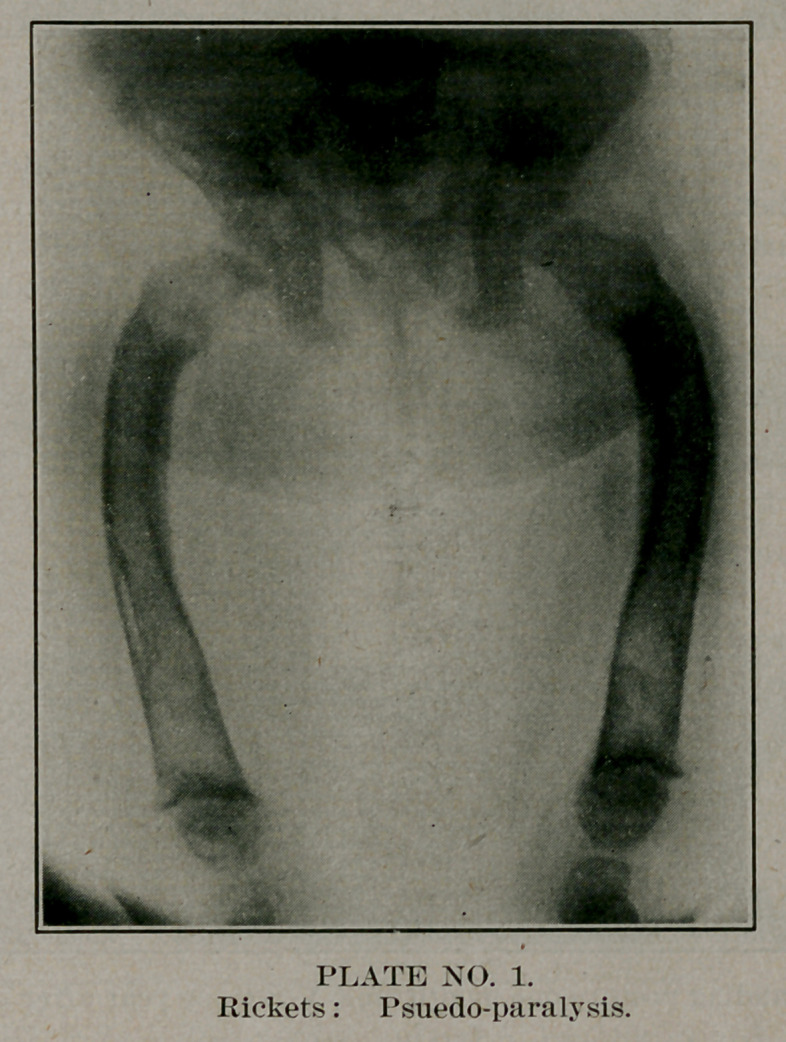


**No. 2. f2:**
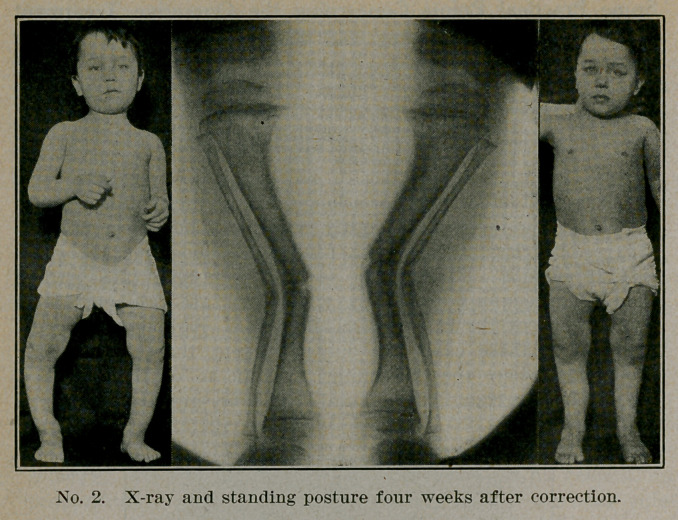


**No. 3. f3:**
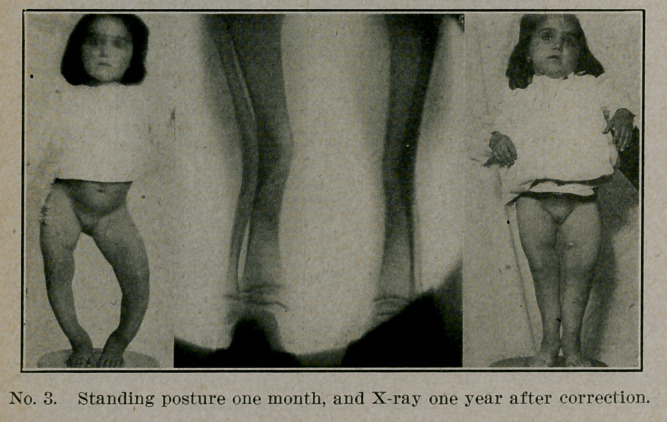


**No. 4. f4:**
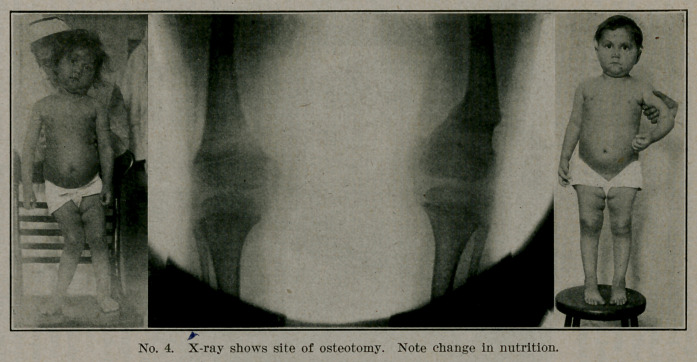


**No. 5. f5:**
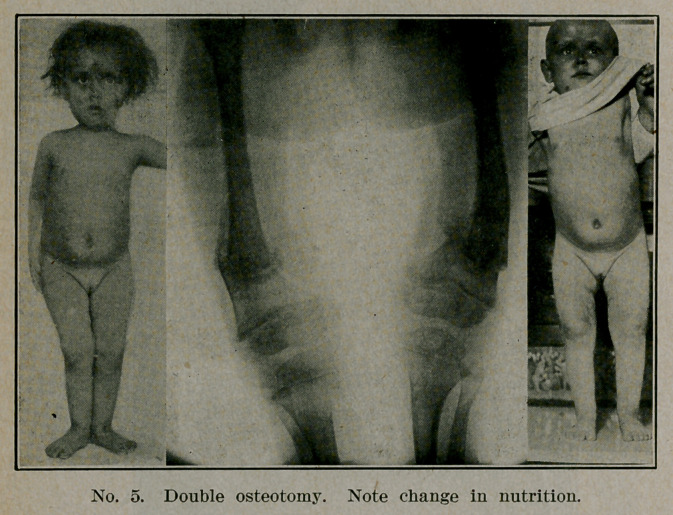


**No. 6. f6:**